# Resveratrol Role in Autoimmune Disease—A Mini-Review

**DOI:** 10.3390/nu9121306

**Published:** 2017-12-01

**Authors:** Ana Lígia de Brito Oliveira, Valter Vinicius Silva Monteiro, Kely Campos Navegantes-Lima, Jordano Ferreira Reis, Rafaelli de Souza Gomes, Dávila Valentina Silva Rodrigues, Silvia Letícia de França Gaspar, Marta Chagas Monteiro

**Affiliations:** 1 Pharmaceutical Science Post-Graduation Program, Neuroscience and Cellular Biology Post Graduation Program, Faculty of Pharmacy, Federal University of Pará, Avenue Augusto Correa SN, Guamá, Pará 66075-110, Brazil; any_015_@hotmail.com (A.L.d.B.O.); kcnavegantes@gmail.com (K.C.N.-L.); rafaelli_gomes@hotmail.com (R.d.S.G.); 2 School of Pharmacy, Health Science Institute, Federal University of Pará/UFPA, Avenue Augusto Correa SN, Guamá, Pará 66075-110, Brazil; valterv@live.com (V.V.S.M.); jordanoreis@outlook.com (J.F.R.); davilavrodrigues@gmail.com (D.V.S.R.); leticiagaspar.farma@hotmail.com (S.L.d.F.G.)

**Keywords:** autoimmunity, resveratrol, inflammation, organ-specific, systemic

## Abstract

Autoimmune diseases are still considered to be pressing concerns due the fact that they are leaders in death and disability causes worldwide. Resveratrol is a polyphenol derived from a variety of foods and beverages, including red grapes and red wine. Anti-inflammatory, antioxidant, and antiaging properties of resveratrol have been reported, and in some animal and human studies this compound reduced and ameliorated the progression of autoimmune diseases, such as rheumatoid arthritis, systemic lupus erythematosus, psoriasis, inflammatory bowel disease, and type 1 diabetes mellitus. Thus, this review aims to summarize and critically analyze the role of resveratrol in the modulation of several organ-specific or systemic autoimmune diseases.

## 1. Introduction

Among chronic diseases, autoimmune diseases are one of the leading causes of morbidity and mortality in the world [1,2]. The main processes regarding their development are failures of the mechanisms of lymphocyte autotolerance, which leads to an imbalance between the activation and regulation of these cells [3]. Dysregulated activation of these lymphocytes can lead to the production of autoantigens, which can cause damage to different tissues. This leads to the predisposition of a local or systemic immune response and inflammation [3].

Development of autoimmune diseases can lead to possible damage to one or more body tissues or organs. Autoimmune diseases can be classified into two types: (1) organ-specific, where the autoimmune process is directed against a single organ and includes diseases such as type 1 diabetes (T1DM), inflammatory bowel diseases (IBD) and psoriasis [4] and (2) systemic autoimmune disorders, where the immune response attacks different organs and tissues at the same time, for example, in rheumatoid arthritis (RA), amyotrophic lateral sclerosis (ALS) and systemic lupus erythematosus (SLE) [5,6].

Natural products have been widely studied in the treatment and prevention of various chronic diseases. Resveratrol—a molecule derived from natural products—is a well-studied substance, known for its effect on a large number of chronic diseases and its high number of therapeutic benefits, that include antioxidant, antiviral, antifungal, cardio protection, and anticancer as well as anti-inflammatory activities [7,8,9,10,11,12,13].

**Figure 1:**
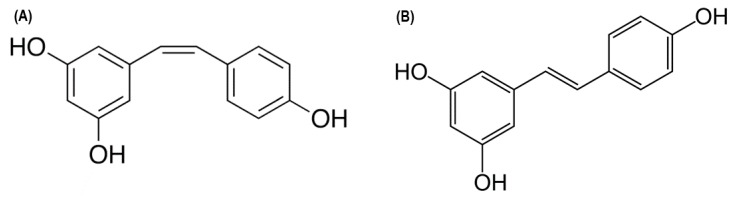
(**A**) cis-resveratrol; (**B**) trans-resveratrol.

Resveratrol is a molecule that has cis-trans isoforms and is chemically known as 3,5,4-trihydroxystilbene (Figure 1). It is a natural phytoalexin synthesized in various plants—grapes, wines, soy, nuts and chocolate [14]—as a response to pathogenic and stressful environmental situations [15]. This molecule is composed of two phenolic rings, one with a double meta hydroxyl substitution and the other with an ortho hydroxyl substitution. Both of them are bound in a non-condensed structure, by a double carbon–carbon bond, which gives this molecule cis-trans isomerism [16] and allows for its distribution to tissues by binding reversibly to serum albumin [17].

The trans isomer of resveratrol is the most abundant and biologically active [18]. However, when the trans isomer is exposed to ultraviolet light, it undergoes photochemical degradation and becomes the less active cis isomer [19]. However, resveratrol is a natural, well-studied substance in most types of chronic diseases, including autoimmune diseases. Thus, this review will detail comprehensive coverage of the current evidence available on the efficacy and possible mechanisms of action of resveratrol in some autoimmune organ-specific and systemic autoimmune diseases, including in vitro assays and animal and human studies. Thus, this work will not only report the strengths but also the limitations of using resveratrol in vivo, since resveratrol has limited bioavailability, due to its rapid in vivo metabolism [20,21]. In addition, this antioxidant also has shown some mild toxic effects during high-dose treatments, such as headache, myalgia of the lower extremities, somnolence, blood electrolyte changes, and rash [22]. Therefore, this review aims to summarize and critically analyze the role of resveratrol as a possible therapeutic option in some organ-specific or systemic autoimmune diseases and consider its effects, bioavailability, and toxicity.

## 2. Effects of Resveratrol on Organ-Specific Autoimmune Diseases

### 2.1. Resveratrol: A Potential T1DM

Similar to other autoimmune diseases, T1DM occurs due the destruction of beta cells in the islets of Langerhans, by autoantigen-specific cluster of differentiation 4 (CD4^+^) and cluster of differentiation 8 (CD8^+^) T lymphocytes [23,24] and by macrophages, dendritic cells (DCs), neutrophils and natural killer T (NKT) cells, which leads inflammation in this tissue [25]. The destruction of beta cells results in insulin deficiency and in hyperglycemia [26,27]. Subsequently, the course of T1DM results in health complications that may include ketoacidosis, kidney failure, heart disease, and blindness [26,28].

**Figure 2:**
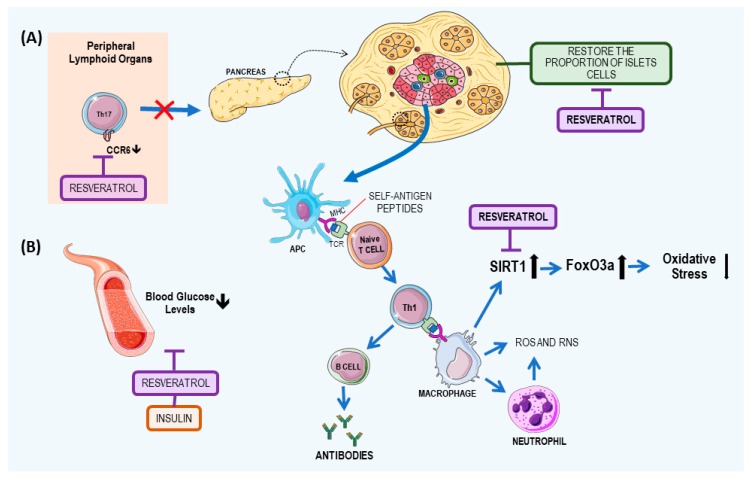
Type 1 diabetes mellitus disease mechanism and resveratrol role. Islet resident dendritic cell (DC) uptake of beta cell antigens, presenting to naïve T cells, and promoting promoting T-helper 1 (Th1) differentiation will activate B lymphocytes that will produce autoantibodies against beta cells. Th1 will also activate macrophage and neutrophil migrations to the islet that will promote beta cell destruction by increasing ROS. Resveratrol acts by (**A**) inhibiting Th1 cell migration by binding to CCR6 and (**B**) forming a complex with insulin that increases its glucose intake. Resveratrol also acts via SIRT1 to inhibit apoptotic cell injury during oxidative stress and increases antioxidant capacity by reducing ROS. In addition, resveratrol also plays a role in restoring beta cells in the islets. CCR6: chemokine receptor 6; APC: antigen presenting cell; FoxO3a: forkhead box O3; MHC: major histocompatibility complex; RNS: reactive nitrogen species; ROS: reactive oxygen species; SIRT1: Sirtuin 1; TCR: T-cell receptor. This figure used elements from Servier Medical Art (www.servier.com).

The process involved in the immunological aspect of the disease is due the inflammatory response. Thus, the release of tumor necrosis factor alpha (TNF-α) and interferon gamma (IFN-γ) by white blood cells stimulates nitric oxide (NO), which interferes with the apoptosis of beta cells and the consequent recruitment of antigen presenting cells (APCs) [29]. APCs activate CD4^+^ T cells, which stimulate macrophages to release cytokines and reactive oxygen species (ROS). The pro-inflammatory environment and the contact with beta cell antigen-specific CD4^+^ T cells can induce the APCs to cross-present antigens to beta cell antigen-specific CD8^+^ T cells, thus enhancing their cytotoxic properties to islet cells [30]. The main mechanisms of development of T1DM and the resveratrol effects on T1DM are summarized on Figure 2.

The pharmacological effects of resveratrol in autoimmunity, especially in T1DM, may be related to the suppression of nuclear factor kappa Β (NF-κB) through the inhibition of other protein and lipid kinases—mitogen-activated protein kinase, plasma creatine kinase, Src family tyrosine kinases, and phosphoinositide 3-kinase (PI3K)—and the suppression of the production of inflammatory cytokines [31].

*In vitro studies.* Another mechanism of resveratrol action is through increased expression of the nicotinamide adenine dinucleotide (NAD) deacetylase-dependent protein sirtuin 1 (SIRT1), which is extensively involved in various physiological processes, by acting as a regulator of the innate and adaptive responses: forkhead transcription factors assigned to the O3a class (FoxO3a), and a lower expression of p47phox, a cytosolic protein in monocytes. FoxO3a is deacetylated by SIRT1, which inhibits apoptotic cell injury during oxidative stress [32,33]. This mechanism was demonstrated by Yun et al. [27], when they evaluated in vitro human monocytes from T1DM patients and acute monocytic leukemia cell line (THP-1). The monocytes were cultured under hyperglycemic conditions (25 mm mol/L), in the presence or absence of resveratrol (3 µmol/L and 6 µmol/L), for 48 h.

*Animal studies.* In an animal model of T1DM, Lee et al. [31] investigated whether resveratrol is capable of prevention and treatment. The authors administered resveratrol orally (250 mg/kg) or by subcutaneous injection (25 mg/kg) and observed that non-obese diabetic mice were protected from T1DM, not only preventing the disease but also reversing higher stages of insulitis in the islets of Langerhans. To elucidate that activity, they observed chemokine receptor 6 (CCR6) protein expression in T cells. The results showed a low amount of CCR6 expression in T helper (Th17) cells and pathogenic CD11b^+^F4/80^hi^ macrophages, blocking the trafficking of the cells from peripheral lymphoid organs to the pancreas, in a manner independent of its ligand chemokine (C–C motif) ligand 20 (CCL20). Another animal study with streptozotocin-induced diabetic rats, administrated 25 mg/kg of resveratrol by gavage and observed that the proportions of acinar and beta cells in the pancreases of healthy animals differed from those in diabetic cells with resveratrol treatment, and they restored the proportion of islet cells with insulin staining [34]. On the other hand, Yonamine et al. [35] evaluated the adjunctive effect of resveratrol (10 mg/kg intraperitoneally) when administrated together with insulin (5 U/day subcutaneously), and they observed a reduction in blood glucose to similar levels observed in non-diabetic rats and a decrement in glycosuria.

### 2.2. Resveratrol as a Supplement to Treat Inflammatory Bowel Disease (IBD)

IBD is a group of clinical manifestations characterized by chronic gastrointestinal inflammation, with Crohn's disease (CD) and ulcerative colitis (UC) being the most common forms [36]. The main forms of the disease can be differentiated through the depth of the inflammation, location, and complications of the disease. Clinically, CD and UC have similar symptoms, including abdominal pain, diarrhea, and hematochezia [37].

IBD does not have a well-defined etiology and is characterized as having a multifactorial and complex pathogenesis. Factors that contribute to the manifestation of the disease include genetic variation, bacterial contamination, and imbalance in the immune system [38]. Many IBD susceptibility genes have been recently identified as being associated with host immune function, including epithelial barrier function and host defense mechanisms in response to pathogens [39]. These genes include intelectin 1 (galactofuranose binding) (*ITLN1*), interleukin 23 receptor (*IL23R*), signal transducer and activator of transcription 3 (*STAT3*) [40], and protein tyrosine phosphatase, non-receptor type (*PTPN2*) [41].

The inadequate inflammatory response to intestinal microorganisms is then immune-mediated in a genetically susceptible host [42,43]. Individuals who have mutations in the gene located on chromosome, *16q12*, that codifies the nucleotide-binding oligomerization domain-containing protein 2 (NOD2) have increased susceptibility to the development of IBD. This gene is responsible for encoding intracellular proteins activated by NF-κB in response to bacterial products [44]. Impaired signaling may result in inadequate mucosal healing and increased permeability in the intestine [37]. In turn, if the acute infection is not resolved by anti-inflammatory mechanisms, the homeostasis of the immune system in the intestine is disrupted, leading to chronic inflammation of the intestine by an excessive response to foreign antigens [39].

The mucosal barrier, when altered, induces the translocation of commensal bacteria and bacterial products from the intestinal lumen to the intestinal wall, which leads to the activation of immune cells [43]. In IBD, dysregulated activation of a subset of the effector T cells—Th1 and Th17—occurs in CD, and in Th2 and Th217 in UC, and leads to an inappropriate inflammatory process. The perpetuation of inflammation in both UC and CD leads to the activation of anti-apoptotic T lymphocyte pathways in the lamina propria of the mucosa with increased interleukin (IL)-17 cytokines [42].

**Figure 3:**
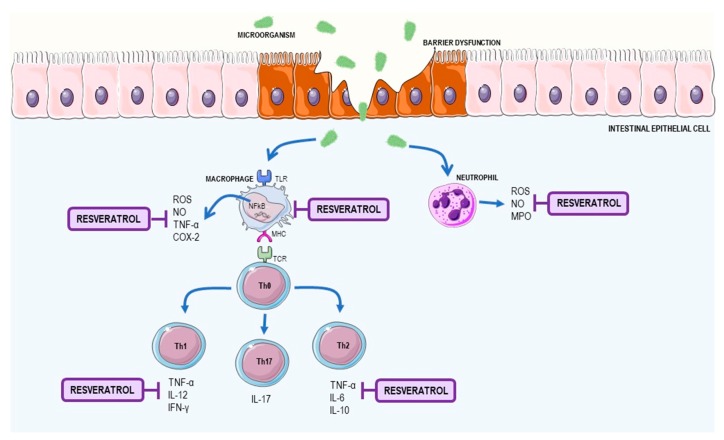
Inflammatory bowel disease (IBD) mechanisms and resveratrol role. IBD develops via a change the intestinal mucosal barrier that leads to a process involving bacterial translocation and subsequent activation of immune cells. The activation of neutrophils and macrophages in the epithelium leads to the production of inflammatory mediators, such as ROS and TNF-α. Antigen recognition by naïve T lymphocytes induces the differentiation to Th1 and Th17 profiles in Crohn’s disease, and to TH2 and Th17 profiles in ulcerative colitis, with the release of inflammatory cytokines, especially TNF-α. Resveratrol is capable of acting on the inhibition of inflammatory cytokines and neutralizing ROS. COX2: cyclooxygenase-2; IFN-γ: interferon gamma; IL: interleukin; MHC: major histocompatibility complex; MPO: myeloperoxidase; NO: nitric oxide; ROS: reactive oxygen species; TCR: T-cell receptor; TNF-α: tumor necrosis factor alpha. This figure used elements from Servier Medical Art (www.servier.com).

After activation of Th1 lymphocytes in CD, release of the IL-12, TNF-α, IFN-γ, and IL-23 cytokines occurs, whereas UC is associated with a Th2 profile, leading to the production of anti-inflammatory cytokines, including IL-4, IL-5, IL-6, and IL-10 [45]. In intestinal inflammation, high levels of TNF-α were found. TNF-α is a key cytokine in the pathogenesis of IBD as it induces cell proliferation and differentiation and is responsible for the upregulation of adhesion molecules in endothelial cells. TNF-α is also associated with apoptosis through the recruitment and autoproteolytic activation of caspases [38]. Overproduction of ROS and reactive nitrogen species (RNS) by macrophages and neutrophils during inflammation of the mucosa can lead to lipid peroxidation and cause changes in protein function, leading to cell death and increased changes in the colonic mucosa [46]. The main mechanisms in the development of IBD and the resveratrol effects on IBD are summarized in Figure 3.

*Animal studies.* Several studies in animal models of IBD have observed an antioxidant and immunomodulatory effect of resveratrol—a reverse in chronic inflammation by reducing inflammatory cytokines as well as reducing reactive species in disorders such as IBD [47,48,49]. Yildiz et al. (2015) evaluated the effect of pre-treatment of resveratrol in rats with trinitrobenzenesulfonic acid (TNBS)-induced colitis. In this study, pre-treatment with resveratrol (10 mg/kg) was able to reduce the microscopic score of tissue and oxidative damage with a reduction in malondialdehyde (MDA) and an increase in glutathione peroxidase (GSH-Px) activity [47]. In a study by Lozano-Pérez et al. (2014), using a trinitrobenzenesulfonic acid model of rat colitis treated with resveratrol, myeloperoxidase activity (MPO) was decreased by the lower infiltration of neutrophils and lower levels of inflammatory markers, such as TNF-α, IL-1β, IL-6, and IL-12, in the rat colitis model [48].

Larrosa et al. (2010) found that resveratrol prodrugs, like resveratrol-3-*O*-(60-*O*-butanoyl)-β-d-glucopyranoside and resveratrol-3-*O*-(60-*O*-octanoyl)-β-d-glucopyranoside, reduced cell damage in rodents with preservation of mucosal architecture and diminished expression of inflammatory cytokines, such as macrophage inflammatory protein 1γ (MIP-1γ) and TNF receptor type I, in a murine model of colon inflammation [49]. In addition, there was an increase in bifidobacteria and lactobacilli, which are correlated with an improvement in intestinal health [49]. In this study, oral pre-treatment, at a dose of 2.1 mg/kg, of prodrugs modulated the mechanism of homeostasis and made the delivery of resveratrol more effective in the colon [49].

Martín and collaborators (2004) verified, during early colonic inflammation in rats, that resveratrol reduced the expression of cyclooxygenase (COX2) and prostaglandin D2 (PGD2) concentrations and also stimulated apoptosis and mucus production in the colon following injury caused by the intracolonic instillation of TNBS [50]. In this study, resveratrol (5–10 mg/kg) gavage treatment, performed at 48, 24 and 1 h prior to the induction of colitis, resulted in improved acute experimental colitis, demonstrating a chemopreventive role of resveratrol in animal models 24 h later [50]. In a study by Sánchez-Fidalgo et al. (2010), the implementation of a diet rich in resveratrol (20 mg/kg) in a dextran sulfate sodium-induced colitis model was able to reduce the concentration of inflammatory cytokines, such as TNF-α and IL-1β, as well as increase levels of the anti-inflammatory cytokine, IL-10. In addition, levels of prostaglandin E synthase-1 (PGES-1), COX2, and inducible nitric oxide synthase (iNOS) were reduced. Likewise, clinical signs of the disease—diarrhea, weight loss, and bleeding—were attenuated in the animals that received this diet [51].

Another study by Rahal et al. (2012) administrated polyphenols by gavage, for 27 days, after the induction of CD, which resulted in a decrease in the prophylactic cytokine transforming growth factor beta 1 (TGF-β1) as well as a series of inflammatory cytokines (IL-1β, IL-6, and TNF-α). In this animal model of CD, induced by peptidoglycan-polysaccharide (PG-PS) or human serum albumin (HSA), they observed that resveratrol decreased inflammation and fibrosis in the intestinal wall of rats [52]. These authors then concluded that resveratrol might be a potent immunomodulatory agent of CD therapy.

*Human studies*. There is still a lack of studies regarding resveratrol in IBD in humans. Samsamikor et al. (2015) verified, in a double-blind trial, that UC patients who took resveratrol supplementation (500 mg/day) for 6 weeks had reduced NF-κB activity in peripheral blood mononuclear cells (PBMC) and reduced plasma levels of TNF-α and high sensitivity C-reactive protein (hs-CRP) [53].

### 2.3. Resveratrol: A Possible Therapeutic Agent for Psoriasis

**Figure 4:**
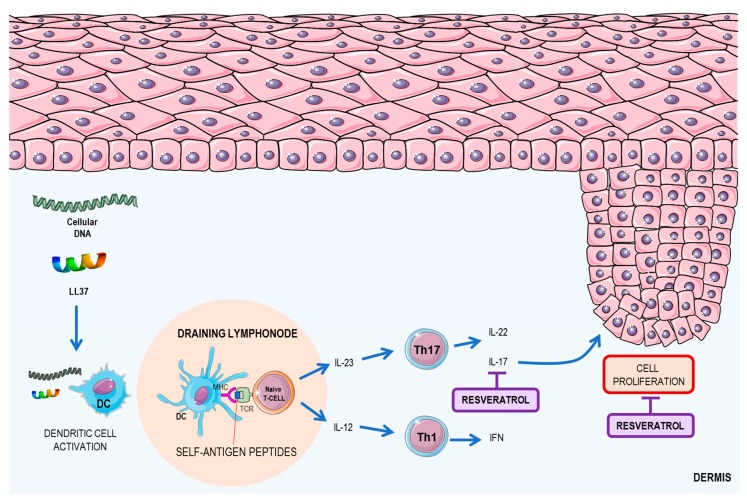
Psoriasis disease mechanism and resveratrol role. Psoriasis begins with the release of cathelicidin peptide (LL37) and fragments of DNA, forming an immunocomplex that activates resident DC cells. These cells activate T lymphocytes primarily through the release of IL-23, promoting differentiation into Th17. IL-16 promotes differentiation into Th1. These cells produce three major cytokines—IL-17, IL-22 (produced by Th17), and IFN (produced by Th1)—that promote the proliferation of keratinocytes. Resveratrol acts in two ways: inhibiting the production of IL-17 and directly inhibiting the proliferation of keratinocytes. DC: dendritic cell; IFN: interferon; IL: interleukin; MHC: major histocompatibility complex; RNS: reactive nitrogen species; ROS: reactive oxygen species; TCR: T-cell receptor. This figure used elements from Servier Medical Art (www.servier.com).

Psoriasis is a genetic disease, trigged by environmental factors, such as infections, injury to the skin, stress, smoking, and alcohol consumption [54]. It is characterized mainly by the appearance of red patches covered with squamous layers. The plaques are characterized by the hyperproliferation of epidermal cells and keratinocytes (including aberrant differentiation of these cells) in addition to the formation of an inflammatory infiltrate in the dermis [54]. This disease is mediated by the immune system (mainly by DCs and T cells) [55,56]. Psoriasis is initiated by conjugation of the cathelicidin peptide LL-37 and self-DNA fragments, originating from keratinocytes, and the posterior presentation of the immune complex by resident DC to naïve T lymphocytes at the draining lymph node [54]. In the chronic phase of the disease, activated resident DCs produce two major cytokines related to psoriasis—IL-12 and IL-23 [55]. Those cytokines promote the T-cell class shift to Th1 and Th17 patterns, respectively, which increases the expression of IL-12 and IFN by Th1 and IL-17 by Th17, which are key cytokines in the development of the disease [57]. The increase in the concentration of these cytokines causes the activation of keratinocytes, which promote cell proliferation and differentiation [56]. The main mechanisms of the development of psoriasis and the resveratrol effects on psoriasis are summarized in Figure 4.

*In vitro studies.* Resveratrol is a molecule that exhibits anti-inflammatory properties, by reducing the production of inflammatory cytokines and promotes keratinocyte cell death through SIRT1 activation. Resveratrol was shown to induce apoptosis in the HaCaT keratinocyte cell line in in vitro studies. The study demonstrated that stimulation with resveratrol could activate the SIRT1 pathway, by increasing its expression, leading to the inhibition of the Protein kinase B (Akt), due to its phosphorylation. This protein plays an important role in regulating cell survival and proliferation [58]. Another study demonstrated that resveratrol was able to inhibit the proliferation of normal human epidermal keratinocytes by inhibiting aquaporin 3 (AQP3), an important cellular survival regulator. This inhibition occurs due to the activation of SIRT1, which leads to increased aryl hydrocarbon receptor nuclear translocator (ARNT) activation, which leads to extracellular signal–regulated kinase (ERK) dephosphorylation, which, in turn, prevents AQP3 activation [59].

*Animal studies.* Using a mouse model of imiquimod-induced psoriasis, a study demonstrated that resveratrol could ameliorate the damage caused by psoriasis, reducing the thickness of the animals' skins as well as decreasing mRNA expression of IL17 and IL19, which are key cytokines in the development of the disease [60]. Another study demonstrated that resveratrol increases the expression of phosphoenolpyruvate carboxykinase 1 (PCK1) and tripartite motif containing 63 (TRIM63)—important proteins related to atrophy and cellular hypertrophy [60].

## 3. Effects of Resveratrol on Systemic Autoimmune Disease

### 3.1. Resveratrol: A Potential Therapeutic Agent for RA

RA is a systemic autoimmune disease that affects 0.5–1% of the population and affects more women than men [61,62,63]. Several factors may contribute to the pathogenesis of the disease—hormonal, environmental, and immunological—that act together in genetically susceptible individuals: human leukocyte antigen (HLA) genes, such as *HLA-DRB1*; encoding protein tyrosine phosphatase non-receptor type 22 (*PTPN22*) gene; peptidyl arginine deiminase type 4 (*PADI4*) gene; and cytotoxic T-lymphocyte associated protein 4 (*CTLA-4*) gene [64].

The pathophysiological mechanism of RA is characterized by chronic inflammation of multiple joints, leading to the destruction of joint cartilage and bone erosion. The pathology of RA includes release of proteolytic enzymes, inflammatory mediators, and reactive species that contribute to worsening clinical symptoms. With persistent inflammation, other organs can also became inflamed, resulting in systemic complications that increase morbidity and mortality, such as vasculitis, cachexia anemia, cerebrovascular issues, lymphoma and depression [65,66].

The immune response involves a sequence of events involving the loss of immunological tolerance of T and B cells against citrullinated autoantigens, resulting in an autoimmune response in the inflamed joint [67]. The activation of T cells results in the activation of macrophages. The cross talk between macrophages and neutrophils in RA was recently discussed. Synovial macrophages may stimulate angiogenesis, leukocyte and lymphocyte recruitment, fibroblast proliferation, and protease secretion, which contribute to cartilage and bone destruction during pannus formation in RA, while neutrophils play a key role in tissue damage and facilitate the inflammatory process [68]. Moreover, endothelial cells that express specific cell adhesion molecules (CAMs) are involved and include E-selectin, intercellular adhesion molecule 1 (ICAM1), and vascular cell adhesion molecule 1 (VCAM1) [66,69].

Many cytokines contribute to RA (IFN-γ, IL-12, IL-21, and IL-23) but the primary cytokines are IL-6, which promotes synovial inflammation, cartilage, and bone destruction; TNF-α, which when dysregulated in experimental animals, is sufficient to cause destructive arthritis and; IL-1, which stimulates synoviocytes and chondrocytes. These cells release matrix metalloproteinases (MPP) and other proteinases that degrade cartilage and bone and increase the expression of COX2 and NO synthase [61,70,71].

**Figure 5:**
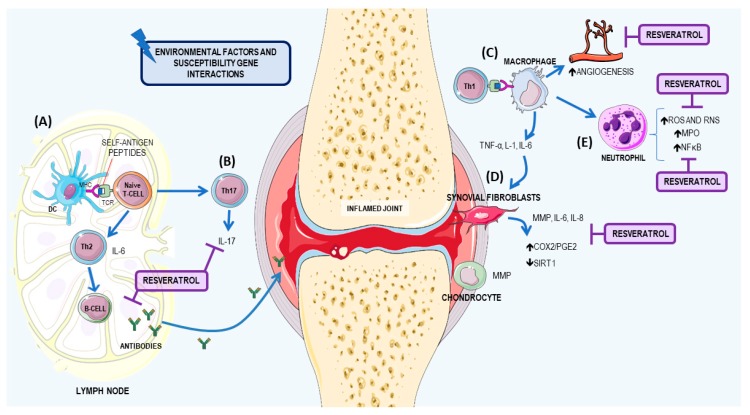
Rheumatoid arthritis mechanism and resveratrol’s role. The pathophysiological mechanism of rheumatoid arthritis (RA) is mediated by environmental factors and susceptible gene interactions, and the immune response involves a sequence of events. (**A**) In the lymph node, antigen recognition occurs by naïve T lymphocytes, which are differentiated to Th2 and Th17, with subsequent activation of B cells, with increased autoantibody production; (**B**) in addition, the Th17 response increases with the pro-inflammatory cytokine, IL-17; (**C**) Synovial macrophages may stimulate angiogenesis, leukocyte and lymphocyte recruitment, fibroblast proliferation, and protease secretion, contributing to cartilage and bone destruction at the site of pannus formation. In addition, the increased cytokine production, especially TNF-α and IL-1, stimulates synoviocytes; (**D**) the synovial fibroblasts at the inflammatory site increase COX2/PGE2, and a decrease in SIRT1. Chondrocytes are also stimulated by synovial macrophages; (**E**) Macrophages increase the recruitment of neutrophils at the inflammatory site, by increasing the production of ROS and RNS and the activation of MPO and NF-κB. Resveratrol is able to act by reducing the production of autoantibodies, Th17 population, oxidative stress and NF-κB activation. Resveratrol also reduces COX2 and PGE2 expression and activates SIRT1, therefore improving the patient's clinical condition. COX2: cyclooxygenase 2; IL: interleukin; TNF-α: tumor necrosis factor alfa; MMP: metalloproteinases; MPO: myeloperoxidase; NF-κB: nuclear factor kappa B; PGE2: prostaglandin E2; ROS: reactive oxygen species; RNS: reactive nitrogen species; SIRT1: sirtuin 1. This figure used elements from Servier Medical Art (www.servier.com).

During the inflammatory process of RA, an increase in ROS occurs, which act as secondary messengers in immunological cellular responses. In addition, free radicals can directly degrade the joint cartilage, attack and inhibit synthesis of proteoglycan, and may also be involved in the mutation of p53 in RA-derived fibroblast-like synoviocytes (FLS) [72,73]. The FLS are the major cells in articular synovial tissues [74]. Recently, studies have demonstrated that SIRTs, such as SIRT1, are involved in the pathogenesis of RA and exhibit abnormal expression in RA synovial tissues [75]. In animal models, SIRT1 deletion aggravates inflammatory arthritis in mice and increases production of pro-inflammatory cytokines in murine macrophages [76]. The main mechanisms for the development of RA and the resveratrol effects on RA are summarized in Figure 5.

In RA, several studies have reported that resveratrol has a potent joint protection effect through the modulation of the inflammatory process with suppression of carrageenan-induced paw edema, chondrolysis, and angiogenesis [77]. The anti-inflammatory effect in RA by resveratrol is through the inhibition of TNF-α and IL-1β induced NF-κB activation, and activator protein 1 (AP1). In addition, it inhibits the enzymatic activities of COX1 and COX2 [78,79].

*In vitro studies.* The literature has many in vitro studies involving human FLS [80,81,82,83]. Recently, Tsai and collaborators (2017) showed, in vitro, that resveratrol stimuli (10 and 20 μM) in human FLS reduced COX2/PGE2 and attenuated nicotinamide adenine dinucleotide phosphate (NADPH) oxidase activity and ROS generation, suggesting that resveratrol could inhibit the inflammatory responses in human FLSs [81]. In another in vitro study, Hao and collaborators (2017) demonstrated that resveratrol treatment (1, 3 and 10 μg/mL) of RA-FLS, collected from patients with RA, who had undergone knee replacement surgery, exhibited increased expression levels of SIRT1 mRNA and protein compared to a control group, in a dose-dependent manner. In addition, resveratrol inhibited the invasive ability of RA-FLS and reduced MPP (mainly MMP9) [82].

Glehr and collaborators (2013) also evaluated the effect of resveratrol in RA-FLS after treatment with 100 μM for 24 h, and they observed inhibition of the overproduction of MMPs and receptor activator of nuclear factor kappa-B ligand (RANKL), which is responsible for causing chondrocyte degeneration and pathological bone resorption [83].

*Animal studies*. Elmali and collaborators (2006) investigated the effects on cartilage destruction and synovial inflammation following intra-articular injections of resveratrol in a rabbit arthritis model. The animals were divided into two groups. The first group received 10 µmol/kg of resveratrol in dimethyl sulfoxide (DMSO), while the second group of animals were treated with DMSO only, under the same protocol as the control group [84]. They observed that the resveratrol group significantly decreased cartilage destruction, had a reduced loss of proteoglycan content in the cartilage, and reduced inflammation.

Riveiro-Naveira and collaborators (2016) used an acute model of antigen-induced arthritis in rats treated with resveratrol (12.5 mg/kg) daily for 2 months by oral gavage. They observed significantly reduced knee swelling, which suggests that oral administration of resveratrol can reduce severity in this model [85]. Chen and collaborators (2014), reported that oral administration of resveratrol (10 or 50 mg/kg body weight) for 2 weeks reversed arthritis dysfunction in adjuvant arthritis rats [86]. In another study by Xuzhu and collaborators (2012), in mice with collagen-induced arthritis (CIA), the authors demonstrated that 20 mg/kg per mouse of resveratrol, had joint-protective properties due to the inhibitory effect on pro-inflammatory cytokine levels, such as IFN-γ, TNF-α, IL-6, IL-1, and IL-4. In addition, this compound reduced the Th17 cell population and the production of IL-17 and autoantibodies [87].

Besides the anti-inflammatory effect, resveratrol is considered a potential agent for RA therapy, because it is an excellent scavenger of hydroxyl, superoxide, and other radicals and improves antioxidant defenses, such superoxide dismutase (SOD), catalase, thioredoxin, and GSH-Px [88,89,90]. Zhang et al. (2016) showed reducing oxidative injury parameters in adjuvant arthritis rats that were treated for 12 days with resveratrol (5, 15, 45 mg/kg) led to a significant reduction in MDA content capacity, glutathione peroxidase, and the glutathione reductase ratio [90]. Wahba, Messiha, and Abo-Saif (2016) also reported that treatment with 10 mg/kg/day of resveratrol in rats with arthritis, induced by complete Freund’s adjuvant (CFA), restored serum MDA and glutathione (GSH) levels back to normal. They also demonstrated an anti-inflammatory effect, through decreased levels of MDA, TNF-α, and MPO [91].

### 3.2. Resveratrol: A Possible Therapeutic Agent to ALS

ALS is a fatal disease that causes selective dysfunction and progressive degeneration of motor neurons [92]. There are two disease classifications: familiar amyotrophic lateral sclerosis (FALS), associated with genetic inheritance, and sporadic ALS (SALS). Both show the same symptoms: muscle weakness and atrophy, paralysis, respiratory insufficiency, and death [92,93].

**Figure 6:**
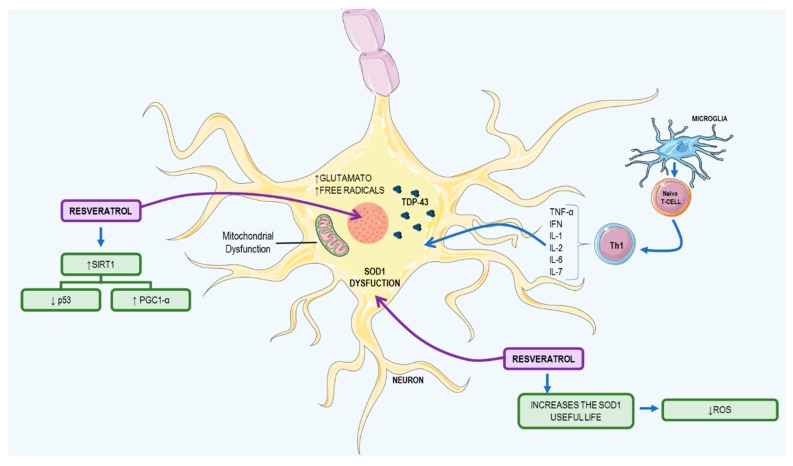
Amyotrophic lateral sclerosis mechanism and resveratrol therapy. TDP-43 accumulation in the motor neuron cytoplasm deregulates mitochondrial biogenesis and SOD1 function, increasing glutamate and free radicals in the cytosol. Microglia detect an abnormal cell and activate naïve T-cell differentiation in the Th1 pattern that releases cytokines (TNF-α, INF, IL-1, IL-2, IL-6, and IL-7). Resveratrol acts by activating SIRT1 and regulates its substrate expression, increases the SOD1 useful life, reduces ROS, and acts in mitochondrial biogenesis as an antioxidant and antiapoptotic. IFN: interferon; IL: interleukin; PGC-1α: peroxisome proliferator-activated receptor gamma coactivator 1-alpha; ROS: reactive oxygen species; SIRT1: sirtuin 1; SOD1: superoxide dismutase 1; TDP-43: TAR DNA-binding protein 43; TNF-α: tumor necrosis factor alpha. This figure used elements from Servier Medical Art (www.servier.com).

This disease occurs due to a mutation in the TAR DNA Binding Protein (*TARDBP*) gene that generates an overexpression of TAR DNA-binding protein 43 (TDP-43) and an enzymatic dysfunction of SOD, an important antioxidant. The TDP-43 protein is responsible for regulating RNA processing and mitochondrial function and when it accumulates in the cytoplasm, it deregulates these processes [94]. On the other hand, aggregation of the SOD enzyme causes mitochondrial damage and increases glutamate and cellular superoxide, increasing free radical production. The microglia recognize autologous antigens of these motor neurons, activate autoreactive CD4^+^ lymphocytes to eliminate the defective cells, and activate CD4^+^ lymphocytes, which generates a Th1 response that increases the secretion of pro-inflammatory cytokines—TNF-α, INF, IL-1 IL-2, IL-6, and IL-7. This event promotes B cell differentiation, which produces specific immunoglobulins (Ig) against self-antigens that trigger a systemic process of cellular destruction [95]. The main mechanisms of development in ALS and the resveratrol effects on ALS are summarized in Figure 6.

*In vitro studies.* In muscle tissue culture cells, studies have shown that a concentration of 50 μM of resveratrol, administered in one or three doses, with a concentration of 20 μM, and administered with a 6-h break, can activate SIRT1, with overexpression of peroxisome proliferator-activated receptor gamma coactivator-1alpha (PGC-1α) and can increase mitochondrial biogenesis over a 24 h period, thus improving motor coordination [96]. In addition, another ALS mouse model has shown that increased PGC-1α expression and activity after resveratrol treatment increases superoxide dismutase 1 (SOD1) life and motor function [97].

*Animal studies.* Studies with Wistar rats and c57BL/6J mice, on a diet of 4 g/kg of resveratrol per day, showed no effect on mitochondrial biogenesis and expression of PGC-1α, because the plasma levels of resveratrol were less than 10 μM. In order to activate mitochondrial biogenesis, the plasma amount must be higher than this concentration [96].

In a study conducted by Song et al. (2014), using the SOD1G93A transgenic mouse model, resveratrol had limited effects, due to its low bioavailability; however, it was capable of inducing the expression of PGC-1α in several tissues, increasing SOD1 lifespan, decreasing p53 activity, and increasing expression of the B-cell lymphoma 2 (BCL2) anti-apoptotic protein. This compound is capable of decreasing motor neuron degeneration and retarding muscular atrophy and has proven to be an efficient antioxidant and antiapoptotic [98].

### 3.3. Resveratrol: A Possible Therapeutic Agent for Systemic Lupus Erythematosus

Lupus is an autoimmune disease, characterized by the production of autoantibodies that recognize components of the cell nucleus, which is the main cause of tissue damage in patients with SLE [99,100]. SLE has complex aspects regarding its pathogenesis, with environmental triggers and a genetic predisposition essential to the onset of the disease. Patients with deficiencies in the early components of complement—especially C1q, C2, and C4—are at risk of developing SLE [101,102]. Moreover, polymorphisms of genes from class II human HLA—DR2 and DR3, mannose binding lectin, T-cell receptor, and IgG Fc receptor—also contribute to the development of SLE in patients [103,104,105]. As for environmental triggers, chemicals, like aromatic amines; drugs, such as hydralazine, isoniazid, and hair dryers; hormonal therapy; and bacterial DNA and endotoxins have been fully associated and reviewed elsewhere [106].

Studies have shown that one possible start to the early stages of SLE and its pathogenesis is related to the impaired clearance of apoptotic cells by macrophages and increased and abnormal apoptosis, which increases the chance of leakage of intracellular antigens that can trigger an autoimmune response, including anti-double stranded DNA. In fact, the presence of anti-double stranded DNA is a common finding in SLE patients [107,108,109]. Altogether, this leads to an increased immune response, with a high activation of T cells, a shift in response from Th1 to Th2, increased IL-10 presence, activation of B cells, and the consequent production of autoantibodies [110,111].

The high amounts of autoantibodies lead to the formation of an immune complex, which can cause damage in peripheral blood vessels, vasculopathy, and vasculitis. These events are common in all affected systems of SLE patients, given that the renal damage and cardiovascular impairment are the most important predictors of mortality [112,113,114]. In a publication by Wang and collaborators (2014), the authors point out that deficiency of SIRT1 is related to the development of autoimmune syndromes, such as lupus in mice, where high titers of antinuclear antibodies and deposition of immunoglobulin in the kidneys can be found [115].

**Figure 7:**
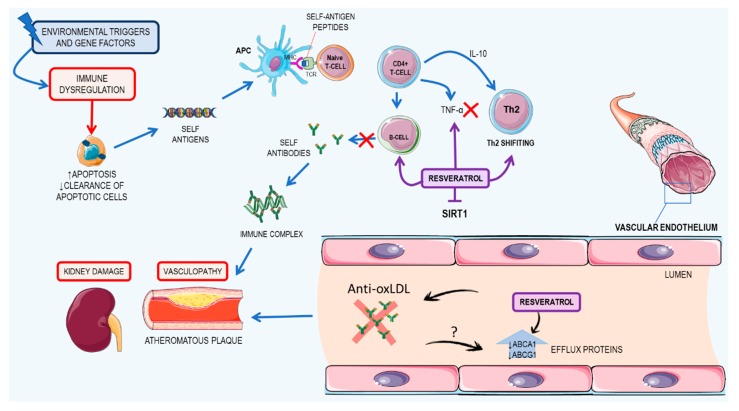
Summary of the pathogenesis of systemic lupus erythematosus (SLE) and resveratrol mechanisms of action. The immune dysregulation caused by environmental triggers and genetic predisposition leads to increased apoptosis. Decreased clearance causes recognition of self-antigens by the immune system, activation of B and T cells, and the production of self-antibodies. Those antibodies, mainly IgG, cause the formation of immune complexes that can lead to renal impairment in kidneys and vasculopathy and atheromatous plaque formation in blood vessels. Atheroma plaques are caused by the action of anti-oxidized low-density lipoprotein (LDL), which is very common in SLE, which leads to the decrease of ABCA1 and ABCG1 by a yet unknown mechanism. Resveratrol acts as an SIRT1 activator, inhibiting proliferation of B and T cells and antibody production and also increasing ABCA1 and ABCG1 levels. ABCA1: ATP-binding cassette transporter A1; ABCG1: ATP-binding cassette transporter G1; APC: antigen presenting cell; IL: interleukin; MHC: major histocompatibility complex; SIRT1: sirtuin 1; TCR: T-cell receptor; TNF-α: tumor necrosis factor alpha. This figure used elements from Servier Medical Art (www.servier.com).

Cardiovascular complications as well as kidney impairment is common in SLE patients and usually contributes to their disability and death. Atherosclerotic cardiovascular disease characteristics in patients with lupus results in the death of 20–30% of said patients [116]. However, the mechanisms of pathogeneses are not fully understood. It is only known that atherosclerosis in SLE patients is facilitated due to the action of IgG antibodies oxidized to lipoproteins, especially low-density lipoprotein (LDL) [117]. Also, Voloshyna and peers (2016) showed that plasma collected from SLE patients had impaired cholesterol flux in vitro [118]. In this regard, this paper will focus on the effects of resveratrol on kidney impairment and cardiovascular complications of SLE. The main mechanisms of development of SLE and the resveratrol effects on SLE are summarized in Figure 7.

#### 3.3.1. Kidney Damage

*Animal studies.* Using in vivo assays to test if resveratrol, a well-known SIRT1 activator, may be useful for SLE treatment, Wang and collaborators (2014) used a pristane-induced lupus BALB/c mouse model [119], in which the mice received an 0.5 mL injection of pristane and were treated with resveratrol (50 mg/kg/day and 75 mg/kg/day) over 7 months and the serum levels of autoantibodies and kidney damage were assessed [115]. They concluded that resveratrol was able to attenuate proteinuria, decrease IgM and IgG kidney deposition, and reduce kidney histological lesions. They also concluded that resveratrol was able to inhibit CD4^+^ T cells and B cell activation in vitro as well as antibody production and B cell proliferation. Also, they found that resveratrol decreased levels of CD4INFy^+^ Th1 cells, the Th1/Th2 ratio, B cells, and Th1 cytokine promoting immunoglobulins (IgG2a, IgG3). All those effects have an unknown mechanism; however, the authors suggested that it may be due to resveratrol SIRT1 activation [120].

Other works have also shown that resveratrol induces the apoptosis of T cells, by Fas, BCL2, BCL2 associated X (BAX), and p53-mediated mechanisms or by depolarizing mitochondrial membranes and activating caspase 9. Resveratrol inhibits COX2 expression, by suppressing NF-κB activation and also inhibits TNF-α-induced inflammation in fibroblasts, by activating SIRT1 [121,122,123,124].

#### 3.3.2. Cardiovascular Impacts

*In vitro studies.* In this regard, in vitro studies have shown that resveratrol positively affects cholesterol transport, reduces the level of oxidized LDL, due to its antioxidant properties, and thus has therapeutic activities in SLE atherosclerotic cardiovascular disease. Yvan-Charvet and colleagues observed that cells treated with 10% SLE plasma had significantly reduced levels of ATP-binding cassette transporter A1 (ABCA1) and ATP-binding cassette transporter G1 (ABCG1)—proteins involved in reverse cholesterol transport and responsible for cholesterol efflux [125]. In comparison to healthy patients, the co-incubation of SLE plasma with resveratrol restored the efflux proteins to cell levels of healthy patients, showing that resveratrol was able to augment the ability of the tested cells to remove cholesterol to the medium.

*Animal studies.* Such results were confirmed in vivo with double knockout ApoE^−^/^−^Fas^−^/^−^ mice—a model that closely represents SLE in mice. They found that the group treated with resveratrol had fewer atherosclerotic plaques in comparison to the untreated group (non-significant), with 43% of the treated animals not developing plaques [117]. They also evaluated the mRNA levels of ABCA1 and ABCG1 in bone marrow-derived macrophages and found that they were significantly higher in the resveratrol-treated group. Both the in vitro and the in vivo tests suggested that resveratrol acts as an antiatherogenic agent by augmenting the cholesterol efflux pathway [117].

Altogether, resveratrol seems to be a potent new drug to treat and delay the progression of SLE regarding kidney and heart failures. However, the lack of data regarding its use in other common SLE complications, such as impairment of lungs, the peripheral nervous system, and the liver, might be an impediment to the commencement of clinical trials for this drug. Also, resveratrol does not seem to have curative properties in SLE and should be treated as an adjuvant in the disease therapy.

## 4. Resveratrol Bioavailability and Toxicity

The major problem faced in the treatment of diseases with resveratrol is in its low bioavailability, where, after oral administration, much of it is metabolized through phase II enzymes, especially glucuronides and sulfatases [21]. Oral studies in humans treated with a single 25 mg/day dose had a peak plasma concentration of 10 ng/mL [126,127]. Studies with high doses of 500 mg/day also had low plasma concentrations of about 71.2 ng/mL [128,129]. Despite the low oral bioavailability, a number of studies have been carried out to try and overcome this problem, such as the creation of prodrugs that, after mentalization, will give rise to resveratrol molecules, or also by microencapsulation through carrier systems [130,131].

As for toxicity, extensive studies using resveratrol supplementation or treatment for an array of diseases have shown some adverse effects in therapeutic approaches, mainly disorders in the gastrointestinal tract and nephrotoxicity with higher doses of supplementation [22,132]. Moreover, several studies have reported that high doses of resveratrol administered orally lead to alterations in the pharmacokinetics of drugs, xenobiotics, and other dietary supplements. These effects are associated with positive or negative modulation of certain cytochrome P450 isozymes (i.e., CYP3A4, CYP1A1 1A2, and CYP2B6, among others) and microsome enzymes, thus interfering in the detoxification or metabolization of these compounds [133,134,135]. Accordingly, Detampel et al. (2012) showed that high doses of resveratrol could inhibit cytochrome P450 isoenzymes, such as CYP3A4, CYP2C9, and CYP2D6, while CYP1A2 is regulated positively by supplementation with resveratrol [136]. These effects become especially risky in conditions where the patient makes concomitant use of resveratrol with drugs or other supplements, such as quercetin and other flavonoids, in order to improve the bioavailability of resveratrol [137]. In this regard, higher doses of resveratrol can compete with other polyphenols for transporters, reducing their uptake and potential synergistic effects [138]. Moreover, individual factors may also influence resveratrol bioavailability and physiological responses, such as variability in the human gut microbiota, genetic polymorphisms, age, sex, race, diet, and exercise practices [139].

On the other hand, recent clinical trials, with low or moderate doses of resveratrol, have shown a positive effect on metabolic parameters and oxidative stress in some chronic diseases and cancer [135,138], as well as several in vitro models of high oxidative stress levels [135,140]. This lack of adverse effects related to low dose resveratrol is due to rapid metabolism and conversion to glucuronate derivative. In addition, signs and symptoms associated with the use of high dose resveratrol disappeared by themselves and showed no sequelae and found high acceptability of resveratrol for the vast majority of tested subjects. In this way, the toxicity of resveratrol was completely covered and reviewed elsewhere [21,133].

## 5. Concluding Remarks

In this review, we included some evidence of antioxidant and immunomodulatory effects for some autoimmune diseases that are directly and indirectly mediated by resveratrol, especially by modulating the immune system and interfering with multiple cellular and molecular processes. Resveratrol is capable of inhibiting T-cell differentiation, especially by inhibiting key cytokines, such as TNF-α, IL-17, IL-6, and IL-1β. This compound is also key in the inhibition of inflammatory transcription factors, such as NF-κB and SIRT1—major regulators of the inflammatory response in some autoimmune diseases. Oxidative stress is other mechanism in which resveratrol is involved. It acts as a direct antioxidant by neutralizing ROS and also improves some antioxidant enzyme activity. In addition, resveratrol also inhibited autoantibody production by plasma cells, which are key factors in the progression of some autoimmune diseases. However, these effects were minimized by the high degree of heterogeneity by the approach of investigators in the in vivo studies, due to the lack of a standard model for these diseases in animals, lack of standardization in the design and duration of treatment, and the lack of agreement on the effective and tolerated dose. In terms of intervention, few studies have addressed the efficacy of resveratrol in autoimmune diseases in humans, making it difficult to obtain concrete evidence of the effect of this antioxidant. Overall, resveratrol appears to be a potent new drug for the therapy of these diseases. However, some barriers have to be overcome, such low bioavailability and adverse effects, as well as the effect of resveratrol administration on patient outcomes. These outcomes are limited by sample size, large range of dosage levels, and various populations and groups studied, although some studies have tried to increase bioavailability by encapsulation with delivery systems. Therefore, more studies and clinical trials should be performed to fully elucidate the beneficial effects of resveratrol supplementation on autoimmunity, as well as its toxic effects on human health.

## References

[1] Lerner A., Jeremias P., Matthias T. (2016). The World Incidence and Prevalence of Autoimmune Diseases is Increasing. Int. J. Celiac Dis..

[2] Cooper G.S., Bynum M.L.K., Somers E.C. (2009). Recent insights in the epidemiology of autoimmune diseases: Improved prevalence estimates and understanding of clustering of diseases. J. Autoimmun..

[3] Rosenblum M.D., Remedios K.A., Abbas A.K. (2015). Mechanisms of human autoimmunity. J. Clin. Investig..

[4] Mastrandrea L.D. (2015). An Overview of Organ-Specific Autoimmune Diseases Including Immunotherapy. Immunol. Investig..

[5] Wahren-Herlenius M., Dorner T. (2013). Immunopathogenic mechanisms of systemic autoimmune disease. Lancet.

[6] Schwartz M., Shechter R. (2010). Systemic inflammatory cells fight off neurodegenerative disease. Nat. Rev. Neurol..

[7] Uzura S., Sekine-suzuki E., Nakanishi I., Sonoda M., Tanimori S. (2016). A facile and rapid access to resveratrol derivatives and their radioprotective activity. Bioorg. Med. Chem. Lett..

[8] Abba Y., Hassim H., Hamzah H., Noordin M.M. (2015). Antiviral Activity of Resveratrol against Human and Animal Viruses. Adv. Virol..

[9] Petrovski G., Gurusamy N., Das D.K. (2011). Resveratrol in cardiovascular health and disease. Ann. N. Y. Acad. Sci..

[10] Farris P., Krutmann J., Li Y.-H., McDaniel D., Krol Y. (2013). Resveratrol: A unique antioxidant offering a multi-mechanistic approach for treating aging skin. J. Drugs Dermatol..

[11] Carter L.G., D’Orazio J.A., Pearson K.J. (2014). Resveratrol and cancer: Focus on in vivo evidence. Endocr. Relat. Cancer.

[12] Das S., Das D.K. (2007). Anti-inflammatory responses of resveratrol. Inflamm. Allergy Drug Targets.

[13] Jung H.J., Hwang I.A., Sung W.S., Kang H., Kang B.S., Seu Y.B., Lee D.G. (2005). Fungicidal effect of resveratrol on human infectious fungi. Arch. Pharm. Res..

[14] Ndiaye M., Kumar R., Ahmad N. (2011). Resveratrol in cancer management: Where are we and where we go from here?. Ann. N. Y. Acad. Sci..

[15] Antus C., Radnai B., Dombovari P., Fonai F., Avar P., Matyus P., Racz B., Sumegi B., Veres B. (2015). Anti-inflammatory effects of a triple-bond resveratrol analog: Structure and function relationship. Eur. J. Pharmacol..

[16] Zarychta B., Gianopoulos C.G., Pinkerton A.A. (2016). Revised structure of trans-resveratrol: Implications for its proposed antioxidant mechanism. Bioorg. Med. Chem. Lett..

[17] Tang F., Xie Y., Cao H., Yang H., Chen X., Xiao J. (2017). Fetal bovine serum influences the stability and bioactivity of resveratrol analogues: A polyphenol-protein interaction approach. Food Chem..

[18] Augustin M.A., Sanguansri L., Lockett T. (2013). Nano- and micro-encapsulated systems for enhancing the delivery of resveratrol. Ann. N. Y. Acad. Sci..

[19] Bonechi C., Martini S., Ciani L., Lamponi S., Rebmann H., Rossi C., Ristori S. (2012). Using liposomes as carriers for polyphenolic compounds: The case of Trans-resveratrol. PLoS ONE.

[20] Pujara N., Jambhrunkar S., Wong K.Y., McGuckin M., Popat A. (2017). Enhanced colloidal stability, solubility and rapid dissolution of resveratrol by nanocomplexation with soy protein isolate. J. Colloid Interface Sci..

[21] Walle T. (2011). Bioavailability of resveratrol. Ann. N. Y. Acad. Sci..

[22] Cottart C.H., Nivet-Antoine V., Laguillier-Morizot C., Beaudeux J.L. (2010). Resveratrol bioavailability and toxicity in humans. Mol. Nutr. Food Res..

[23] Battaglia M. (2014). Neutrophils and type 1 autoimmune diabetes. Curr. Opin. Hematol..

[24] Werstuck G.H., Cheema S.K. (2006). Molecular and cellular mechanisms by which diabetes mellitus promotes the development of atherosclerosis. Biochemistry of Atherosclerosis.

[25] Vives-Pi M., Rodríguez-Fernández S., Pujol-Autonell I. (2015). How apoptotic β-cells direct immune response to tolerance or to autoimmune diabetes: A review. Apoptosis.

[26] Skyler J.S., Bakris G.L., Bonifacio E., Darsow T., Eckel R.H., Groop L., Groop P.-H., Handelsman Y., Insel R.A., Mathieu C. (2017). Differentiation of Diabetes by Pathophysiology, Natural History, and Prognosis. Diabetes.

[27] You S., Chatenoud L. (2016). Autoimmune Diabetes: An Overview of Experimental Models and Novel Therapeutics. Methods Mol. Biol..

[28] Todd J.A. (2010). Etiology of Type 1 Diabetes. Immunity.

[29] Cnop M., Welsh N., Jonas J.-C., Jorns A., Lenzen S., Eizirik D.L. (2005). Mechanisms of pancreatic beta-cell death in type 1 and type 2 diabetes: Many differences, few similarities. Diabetes.

[30] Wallberg M., Cooke A. (2013). Immune mechanisms in type 1 diabetes. Trends Immunol..

[31] Lee S.M., Yang H., Tartar D.M., Gao B., Luo X., Ye S.Q., Zaghouani H., Fang D. (2011). Prevention and treatment of diabetes with resveratrol in a non-obese mouse model of type 1 diabetes. Diabetologia.

[32] Jagani Z., Singh A., Khosravi-Far R. (2008). FoxO tumor suppressors and BCR-ABL-induced leukemia: A matter of evasion of apoptosis. Biochim. Biophys. Acta.

[33] Van der Horst A., Burgering B.M.T. (2007). Stressing the role of FoxO proteins in lifespan and disease. Nat. Rev. Mol. Cell Biol..

[34] Kaur G., Padiya R., Adela R., Putcha U.K., Reddy G.S., Reddy B.R., Kumar K.P., Chakravarty S., Banerjee S.K. (2016). Garlic and resveratrol attenuate diabetic complications, loss of β-cells, pancreatic and hepatic oxidative stress in streptozotocin-induced diabetic rats. Front. Pharmacol..

[35] Yonamine C.Y., Pinheiro-Machado E., Michalani M.L., Freitas H.S., Okamoto M.M., Corrêa-Giannella M.L., Machado U.F. (2016). Resveratrol improves glycemic control in insulin-treated diabetic rats: Participation of the hepatic territory. Nutr. Metab..

[36] Young Hong M. (2014). Effects of Resveratrol on Inflammatory Bowel Disease: A Review. J. Nutr. Health Food Sci..

[37] Kim D.H., Cheon J.H. (2017). Pathogenesis of Inflammatory Bowel Disease and Recent Advances in Biologic Therapies. Immune Netw..

[38] Fakhoury M., Negrulj R., Mooranian A., Al-Salami H. (2014). Inflammatory bowel disease: Clinical aspects and treatments. J. Inflamm. Res..

[39] Hisamatsu T., Kanai T., Mikami Y., Yoneno K., Matsuoka K., Hibi T. (2013). Immune aspects of the pathogenesis of inflammatory bowel disease. Pharmacol. Ther..

[40] Mcgovern D.P.B., Gardet A., Törkvist L., Goyette P., Essers J., Taylor K.D., Neale B.M., Ong R.T.H., Lagacé C., Li C. (2010). Genome-wide association identifies multiple ulcerative colitis susceptibility loci. Nat. Genet..

[41] Wiede F., Shields B.J., Chew S.H., Kyparissoudis K., Vliet C., Van Galic S., Tremblay M.L., Russell S.M., Godfrey D.I., Tiganis T. (2011). T cell protein tyrosine phosphatase attenuates T cell signaling to maintain tolerance in mice. J. Clin. Invest..

[42] Boirivant M., Cossu A. (2012). Inflammatory bowel disease. Oral Dis..

[43] Neurath M.F. (2014). Cytokines in inflammatory bowel disease. Nat. Rev. Immunol..

[44] Ogura Y., Bonen D.K., Inohara N., Nicolae D.L., Chen F.F., Ramos R., Britton H., Moran T., Karaliuskas R., Duerr R.H. (2001). A frameshift mutation in NOD2 associated with susceptibility to Crohn’s disease. Nature.

[45] Singh U.P., Singh N.P., Brandon B., Guan H., Singh B., Price R.L., Taub D.D., Mishra M.K., Nagarkatti M., Nagarkatti P.S. (2012). Alternative Medicines as Emerging Therapies for Inflammatory Bowel Diseases. Int. Rev. Immunol..

[46] Tian T., Wang Z., Zhang J. (2017). Pathomechanisms of Oxidative Stress in Inflammatory Bowel Disease and Potential Antioxidant Therapies. Oxid. Med. Cell. Longev..

[47] Yildiz G., Yildiz Y., Ulutas P.A., Yaylali A., Ural M. (2015). Resveratrol Pretreatment Ameliorates TNBS Colitis in Rats. Recent Pat. Endocr. Metab. Immune Drug Discov..

[48] Lozano-Pérez A.A., Rodriguez-Nogales A., Ortiz-cullera V., Algieri F., Garrido-Mesa J., Zorrilla P., Rodriguez-Cabezas M.E., Garrido-Mesa N., Utrilla M.P., De Matteis L. (2014). Silk fibroin nanoparticles constitute a vector for controlled release of resveratrol in an experimental model of inflammatory bowel disease in rats. Int. J. Nanomed..

[49] Larrosa M., Tomé-Carneiro J., Yáñez-Gascón M.J., Alcántara D., Selma M.V., Beltrán D., García-Conesa M.T., Urbán C., Lucas R., Tomás-Barberán F. (2010). Preventive oral treatment with resveratrol pro-prodrugs drastically reduce colon inflammation in rodents. J. Med. Chem..

[50] Martín A.R., Villegas I., La Casa C., De La Lastra C.A. (2004). Resveratrol, a polyphenol found in grapes, suppresses oxidative damage and stimulates apoptosis during early colonic inflammation in rats. Biochem. Pharmacol..

[51] Sánchez-Fidalgo S., Cárdeno A., Villegas I., Talero E., de la Lastra C.A. (2010). Dietary supplementation of resveratrol attenuates chronic colonic inflammation in mice. Eur. J. Pharmacol..

[52] Rahal K., Schmiedlin-Ren P., Adler J., Dhanani M., Sultani V., Rittershaus A.C., Reingold L., Zhu J., Mckenna B.J., Christman G.M. (2012). Resveratrol has antiinflammatory and antifibrotic effects in the peptidoglycan-polysaccharide rat model of Crohn’s disease. Inflamm. Bowel Dis..

[53] Samsamikor M., Daryani E., Asl R., Hekmatdoost A. (2015). Anti-Inflammatory Effects of Resveratrol in Patients with Ulcerative Colitis : A Randomized, Double-Blind, Placebo-controlled Pilot Study. Arch. Med. Res..

[54] Boehncke W.-H., Schön M.P. (2015). Psoriasis. Lancet.

[55] Hansel A., Gunther C., Ingwersen J., Starke J., Schmitz M., Bachmann M., Meurer M., Rieber E.P., Schakel K. (2011). Human slan (6-sulfo LacNAc) dendritic cells are inflammatory dermal dendritic cells in psoriasis and drive strong TH17/TH1 T-cell responses. J. Allergy Clin. Immunol..

[56] Lowes M.A., Suarez-Farinas M., Krueger J.G. (2014). Immunology of psoriasis. Annu. Rev. Immunol..

[57] Lynde C.W., Poulin Y., Vender R., Bourcier M., Khalil S. (2014). Interleukin 17A: Toward a new understanding of psoriasis pathogenesis. J. Am. Acad. Dermatol..

[58] Lee J.H., Kim J.S., Park S.Y., Lee Y.J. (2016). Resveratrol induces human keratinocyte damage via the activation of class III histone deacetylase, Sirt1. Oncol. Rep..

[59] Wu Z., Uchi H., Morino-Koga S., Shi W., Furue M. (2014). Resveratrol inhibition of human keratinocyte proliferation via SIRT1/ARNT/ERK dependent downregulation of aquaporin 3. J. Dermatol. Sci..

[60] Kjær T.N., Thorsen K., Jessen N., Stenderup K., Pedersen S.B. (2015). Resveratrol ameliorates imiquimod-induced psoriasis-like skin inflammation in mice. PLoS ONE.

[61] Krause M.L., Makol A. (2016). Management of rheumatoid arthritis during pregnancy: Challenges and solutions. Open Access Rheumatol..

[62] Gibofsky A. (2012). Overview of epidemiology, pathophysiology, and diagnosis of rheumatoid arthritis. Am. J. Manag. Care.

[63] Bellucci E., Terenzi R., La Paglia G.M.C., Gentileschi S., Tripoli A., Tani C., Alunno A. (2016). One year in review 2016: Pathogenesis of rheumatoid arthritis. Clin. Exp. Rheumatol..

[64] Smolen J.S., Aletaha D., McInnes I.B. (2016). Rheumatoid arthritis. Lancet.

[65] Anic B., Mayer M. (2014). Pathogenesis of rheumatoid arthritis. Reumatizam.

[66] Mellado M., Martínez-Muñoz L., Cascio G., Lucas P., Pablos J.L., Rodríguez-Frade J.M. (2015). T cell migration in rheumatoid arthritis. Front. Immunol..

[67] Scott D.L., Wolfe F., Huizinga T.W.J. (2010). Rheumatoid arthritis. Lancet.

[68] Navegantes K.C., de Souza Gomes R., Pereira P.A.T., Czaikoski P.G., Azevedo C.H.M., Monteiro M.C. (2017). Immune modulation of some autoimmune diseases: The critical role of macrophages and neutrophils in the innate and adaptive immunity. J. Transl. Med..

[69] Kay J., Calabrese L. (2004). The role of interleukin-1 in the pathogenesis of rheumatoid arthritis. Rheumatology.

[70] Tanaka T., Hishitani Y., Ogata A. (2014). Monoclonal antibodies in rheumatoid arthritis: Comparative effectiveness of tocilizumab with tumor necrosis factor inhibitors. Biol. Targets Ther..

[71] Brzustewicz E., Bryl E. (2015). The role of cytokines in the pathogenesis of rheumatoid arthritis - Practical and potential application of cytokines as biomarkers and targets of personalized therapy. Cytokine.

[72] Quinonez-Flores C.M., Gonzalez-Chavez S.A., Del Rio Najera D., Pacheco-Tena C. (2016). Oxidative Stress Relevance in the Pathogenesis of the Rheumatoid Arthritis: A Systematic Review. BioMed Res. Int..

[73] Hadjigogos K. (2003). The role of free radicals in the pathogenesis of rheumatoid arthritis. Panminerva Med..

[74] Li D., Xiao Z., Wang G., Song X. (2015). Knockdown of ADAM10 inhibits migration and invasion of fibroblast-like synoviocytes in rheumatoid arthritis. Mol. Med. Rep..

[75] Niederer F., Ospelt C., Brentano F., Hottiger M.O., Gay R.E., Gay S., Detmar M., Kyburz D. (2011). SIRT1 overexpression in the rheumatoid arthritis synovium contributes to proinflammatory cytokine production and apoptosis resistance. Ann. Rheum. Dis..

[76] Engler A., Tange C., Frank-Bertoncelj M., Gay R.E., Gay S., Ospelt C. (2016). Regulation and function of SIRT1 in rheumatoid arthritis synovial fibroblasts. J. Mol. Med..

[77] Nguyen C., Savouret J.-F., Widerak M., Corvol M.-T., Rannou F. (2017). Resveratrol, Potential Therapeutic Interest in Joint Disorders: A Critical Narrative Review. Nutrients.

[78] Ma C., Wang Y., Dong L., Li M., Cai W. (2015). Anti-inflammatory effect of resveratrol through the suppression of NF-kappaB and JAK/STAT signaling pathways. Acta Biochim. Biophys. Sin..

[79] Lee S.J., Thien Quach C.H., Jung K.-H., Paik J.-Y., Lee J.H., Park J.W., Lee K.-H. (2014). Oxidized low-density lipoprotein stimulates macrophage 18F-FDG uptake via hypoxia-inducible factor-1alpha activation through Nox2-dependent reactive oxygen species generation. J. Nucl. Med..

[80] Tian J., Chen J., Gao J., Li L., Xie X. (2013). Resveratrol inhibits TNF-α-induced IL-1β, MMP-3 production in human rheumatoid arthritis fibroblast-like synoviocytes via modulation of PI3kinase/Akt pathway. Rheumatol. Int..

[81] Tsai M.-H., Hsu L.-F., Lee C.-W., Chiang Y.-C., Lee M.-H., How J.-M., Wu C.-M., Huang C.-L., Lee I.-T. (2017). Resveratrol inhibits urban particulate matter-induced COX-2/PGE2 release in human fibroblast-like synoviocytes via the inhibition of activation of NADPH oxidase/ROS/NF-kappaB. Int. J. Biochem. Cell Biol..

[82] Hao L., Wan Y., Xiao J., Tang Q., Deng H., Chen L. (2017). A study of Sirt1 regulation and the effect of resveratrol on synoviocyte invasion and associated joint destruction in rheumatoid arthritis. Mol. Med. Rep..

[83] Glehr M., Fritsch-Breisach M., Lohberger B., Walzer S.M., Moazedi-Fuerst F., Rinner B., Gruber G., Graninger W., Leithner A., Windhager R. (2013). Influence of resveratrol on rheumatoid fibroblast-like synoviocytes analysed with gene chip transcription. Phytomedicine.

[84] Elmali N., Baysal O., Harma A., Esenkaya I., Mizrak B. (2006). Effects of resveratrol in inflammatory arthritis. Inflammation.

[85] Riveiro-Naveira R.R., Valcárcel-Ares M.N., Almonte-Becerril M., Vaamonde-García C., Loureiro J., Hermida-Carballo L., López-Peláez E., Blanco F.J., López-Armada M.J. (2016). Resveratrol lowers synovial hyperplasia, inflammatory markers and oxidative damage in an acute antigen-induced arthritis model. Rheumatology.

[86] Chen X., Lu J., An M., Ma Z., Zong H., Yang J. (2014). Anti-inflammatory effect of resveratrol on adjuvant arthritis rats with abnormal immunological function via the reduction of cyclooxygenase-2 and prostaglandin E2. Mol. Med. Rep..

[87] Xuzhu G., Komai-Koma M., Leung B.P., Howe H.S., McSharry C., McInnes I.B., Xu D. (2012). Resveratrol modulates murine collagen-induced arthritis by inhibiting Th17 and B-cell function. Ann. Rheum. Dis..

[88] Diaz-Gerevini G.T., Repossi G., Dain A., Tarres M.C., Das U.N., Eynard A.R. (2016). Beneficial action of resveratrol: How and why?. Nutrition.

[89] Carrizzo A., Puca A., Damato A., Marino M., Franco E., Pompeo F., Traficante A., Civitillo F., Santini L., Trimarco V. (2013). Resveratrol improves vascular function in patients with hypertension and dyslipidemia by modulating NO metabolism. Hypertension.

[90] Zhang J., Song X., Cao W., Lu J., Wang X., Wang G., Wang Z., Chen X. (2016). Autophagy and mitochondrial dysfunction in adjuvant-arthritis rats treatment with resveratrol. Sci. Rep..

[91] Wahba M.G.F., Messiha B.A.S., Abo-Saif A.A. (2015). Protective effects of fenofibrate and resveratrol in an aggressive model of rheumatoid arthritis in rats. Pharm. Biol..

[92] Katz J.S., Dimachkie M.M., Barohn R.J. (2015). Amyotrophic Lateral Sclerosis: A Historical Perspective. Neurol. Clin..

[93] Saberi S., Stauffer J.E., Schulte D.J., Ravits J. (2015). Neuropathology of Amyotrophic Lateral Sclerosis and Its Variants. Neurol. Clin..

[94] Avendano-Vazquez S.E., Dhir A., Bembich S., Buratti E., Proudfoot N., Baralle F.E. (2012). Autoregulation of TDP-43 mRNA levels involves interplay between transcription, splicing, and alternative polyA site selection. Genes Dev..

[95] Malaspina A., Puentes F., Amor S. (2015). Disease origin and progression in amyotrophic lateral sclerosis: An immunology perspective. Int. Immunol..

[96] Higashida K., Kim S.H., Jung S.R., Asaka M., Holloszy J.O., Han D.H. (2013). Effects of Resveratrol and SIRT1 on PGC-1α Activity and Mitochondrial Biogenesis: A Reevaluation. PLoS Biol..

[97] Zhao W., Varghese M., Yemul S., Pan Y., Cheng A., Marano P., Hassan S., Vempati P., Chen F., Qian X. (2011). Peroxisome proliferator activator receptor gamma coactivator-1alpha (PGC-1α) improves motor performance and survival in a mouse model of amyotrophic lateral sclerosis. Mol. Neurodegener..

[98] Song L., Chen L., Zhang X., Li J., Le W. (2014). Resveratrol ameliorates motor neuron degeneration and improves survival in SOD1G93A mouse model of amyotrophic lateral sclerosis. BioMed Res. Int..

[99] Sestak A.L., Nath S.K., Sawalha A.H., Harley J.B. (2007). Current status of lupus genetics. Arthritis Res.Ther..

[100] Rahman A., Isenberg D.A. (2008). Systemic Lupus Erythematosus. N. Engl. J. Med..

[101] Walport M., Davies K., Botto M. (1998). C1q and systemic lupus erythematosus. Immunobiology.

[102] Atkinson J. (1986). Complement activation and complement receptors in systemic lupus erythematosus. Springer Semin. Immunopathol..

[103] Schur P. (1995). Genetics of systemic lupus erythematosus. Lupus.

[104] Ho Lee Y., Witte T., Momot T., Schmidt R.E., Kaufman K.M., Harley J.B., Sestak A.L. (2005). The mannose-binding lectin gene polymorphisms and systemic lupus erythematosus: Two case-control studies and a meta-analysis. Arthritis Rheum..

[105] Pradhan V., Surve P., Rajadhyaksha A., Rajendran V., Patwardhan M., Umare V., Ghosh K., Nadkarni A. (2015). Mannose binding lectin (MBL) 2 gene polymorphism and its association with clinical manifestations in systemic lupus erythematosus (SLE) patients from western India. Indian J. Med. Res..

[106] Mok C.C., Lau C.S. (2003). Pathogenesis of systemic lupus erythematosus. J. Clin. Pathol..

[107] Tan E.M., Cohen A.S., Fries J.F., Masi A.T., Mcshane D.J., Rothfield N.F., Schaller J.G., Talal N., Winchester R.J. (1982). The 1982 revised criteria for the classification of systemic lupus erythematosus. Arthritis Rheum..

[108] Herrmann M., Voll R.E., Zoller O.M., Hagenhofer M., Ponner B.B., Kalden J.R. (1998). Impaired phagocytosis of apoptotic cell material by monocyte-derived macrophages from patients with systemic lupus erythematosus. Arthritis Rheum..

[109] Deng S.X., Hanson E., Sanz I. (2000). In vivo cell penetration and intracellular transport of anti-Sm and anti-La autoantibodies. Int. Immunol..

[110] Klinman D.M., Shirai A., Ishigatsubo Y., Conover J., Steinberg A.D. (2010). Quantitation of IgM- and IgG-secreting B cells in the peripheral blood of patients with systemic lupus erythematosus. Arthritis Rheum..

[111] Houssiau F.A., Lefebvre C., Vanden Berghe M., Lambert M., Devogelaer J.-P., Renauld J.-C. (1995). Serum interleukin 10 titers in systemic lupus erythematosus reflect disease activity. Lupus.

[112] Danila M.I., Pons-Estel G.J., Zhang J., Vilá L.M., Reveille J.D., Alarcón G.S. (2009). Renal damage is the most important predictor of mortality within the damage index: Data from LUMINA LXIV, a multiethnic US cohort. Rheumatology.

[113] Westerweel P.E., Luyten R.K.M.A.C., Koomans H.A., Derksen R.H.W.M., Verhaar M.C. (2007). Premature atherosclerotic cardiovascular disease in systemic lupus erythematosus. Arthritis Rheum..

[114] Kahlenberg J.M., Kaplan M.J. (2013). Mechanisms of premature atherosclerosis in rheumatoid arthritis and lupus. Annu. Rev. Med..

[115] Wang Z.L., Luo X.F., Li M.T., Xu D., Zhou S., Chen H.Z., Gao N., Chen Z., Zhang L.L., Zeng X.F. (2014). Resveratrol possesses protective effects in a pristane-induced lupus mouse model. PLoS ONE.

[116] Lockshin M.D., Salmon J.E., Roman M.J. (2001). Atherosclerosis and lupus: A work in progress. Arthritis Rheum..

[117] Feng X., Li H., Rumbin A.A., Wang X., La Cava A., Brechtelsbauer K., Castellani L.W., Witztum J.L., Lusis A.J., Tsao B.P. (2007). ApoE^−/−^ Fas^−/−^ C57BL/6 mice: A novel murine model simultaneously exhibits lupus nephritis, atherosclerosis, and osteopenia. J. Lipid Res..

[118] Voloshyna I., Teboul I., Littlefield M.J., Siegart N.M., Turi G.K., Fazzari M.J., Carsons S.E., DeLeon J., Reiss A.B. (2016). Resveratrol counters systemic lupus erythematosus-associated atherogenicity by normalizing cholesterol efflux. Exp. Biol. Med..

[119] Satoh M., Reeves W.H. (1994). Induction of lupus-associated autoantibodies in BALB/c mice by intraperitoneal injection of pristane. J. Exp. Med..

[120] Nakata R., Takahashi S., Inoue H. (2012). Recent Advances in the Study on Resveratrol. Biol. Pharm. Bull..

[121] Ko Y.-C., Chang C.-L., Chien H.-F., Wu C.-H., Lin L.-I. (2011). Resveratrol enhances the expression of death receptor Fas/CD95 and induces differentiation and apoptosis in anaplastic large-cell lymphoma cells. Cancer Lett..

[122] Dörrie J., Gerauer H., Wachter Y., Zunino S.J. (2001). Resveratrol induces extensive apoptosis by depolarizing mitochondrial membranes and activating caspase-9 in acute lymphoblastic leukemia cells. Cancer Res..

[123] Roman V., Billard C., Kern C., Ferry-Dumazet H., Izard J.C., Mohammad R., Mossalayi D.M., Kolb J.P. (2002). Analysis of resveratrol-induced apoptosis in human B-cell chronic leukaemia. Br. J. Haematol..

[124] Zhu X., Liu Q., Wang M., Liang M., Yang X., Xu X., Zou H., Qiu J. (2011). Activation of Sirt1 by Resveratrol Inhibits TNF-α Induced Inflammation in Fibroblasts. PLoS ONE.

[125] Yvan-Charvet L., Wang N., Tall A.R. (2010). Role of HDL, ABCA1 and ABCG1 transporters in cholesterol efflux and immune responses. Arterioscler. Thromb. Vasc. Biol..

[126] Walle T., Hsieh F., DeLegge M.H., Oatis J.E.J., Walle U.K. (2004). High absorption but very low bioavailability of oral resveratrol in humans. Drug Metab. Dispos..

[127] Goldberg D.M., Yan J., Soleas G.J. (2003). Absorption of three wine-related polyphenols in three different matrices by healthy subjects. Clin. Biochem..

[128] Almeida L., Vaz-da-Silva M., Falcao A., Soares E., Costa R., Loureiro A.I., Fernandes-Lopes C., Rocha J.-F., Nunes T., Wright L. (2009). Pharmacokinetic and safety profile of trans-resveratrol in a rising multiple-dose study in healthy volunteers. Mol. Nutr. Food Res..

[129] Sergides C., Chirilă M., Silvestro L., Pitta D., Pittas A. (2016). Bioavailability and safety study of resveratrol 500 mg tablets in healthy male and female volunteers. Exp. Ther. Med..

[130] Biasutto L., Mattarei A., Azzolini M., La Spina M., Sassi N., Romio M., Paradisi C., Zoratti M. (2017). Resveratrol derivatives as a pharmacological tool. Ann. N. Y. Acad. Sci..

[131] Davidov-Pardo G., McClements D.J. (2014). Resveratrol encapsulation: Designing delivery systems to overcome solubility, stability and bioavailability issues. Trends Food Sci. Technol..

[132] Cottart C.H., Nivet-Antoine V., Beaudeux J.L. (2013). Review of recent data on the metabolism, biological effects, and toxicity of resveratrol in humans. Mol. Nutr. Food Res..

[133] Wahab A., Gao K., Jia C., Zhang F., Tian G., Murtaza G., Chen J. (2017). Significance of Resveratrol in Clinical Management of Chronic Diseases. Molecules.

[134] MacDonald L., Foster B.C., Akhtar H. (2009). Food and therapeutic product interactions—A therapeutic perspective. J. Pharm. Pharm. Sci..

[135] Guthrie A.R., Chow H.-H.S., Martinez J.A. (2017). Effects of resveratrol on drug- and carcinogen-metabolizing enzymes, implications for cancer prevention. Pharmacol. Res. Perspect..

[136] Detampel P., Beck M., Krahenbuhl S., Huwyler J. (2012). Drug interaction potential of resveratrol. Drug Metab. Rev..

[137] De Santi C., Pietrabissa A., Spisni R., Mosca F., Pacifici G.M. (2000). Sulphation of resveratrol, a natural compound present in wine, and its inhibition by natural flavonoids. Xenobiotica.

[138] Leon D., Uribe E., Zambrano A., Salas M. (2017). Implications of Resveratrol on Glucose Uptake and Metabolism. Molecules.

[139] Novelle M.G., Wahl D., Dieguez C., Bernier M., de Cabo R. (2015). Resveratrol supplementation: Where are we now and where should we go?. Ageing Res. Rev..

[140] Albuquerque R.V., Malcher N.S., Amado L.L., Coleman M.D., Dos Santos D.C., Borges R.S., Valente S.A.S., Valente V.C., Monteiro M.C. (2015). In Vitro Protective Effect and Antioxidant Mechanism of Resveratrol Induced by Dapsone Hydroxylamine in Human Cells. PLoS ONE.

